# Psychosocial aspects in temporomandibular disorder: clinical case report

**DOI:** 10.1080/07853890.2021.1896646

**Published:** 2021-09-28

**Authors:** Paula Moleirinho-Alves, Pedro Cebola, André Mariz, Almeida

**Affiliations:** a Escola Superior de Saúde Egas Moniz (ESSEM) , Egas Moniz Cooperativa de Ensino Superior, Caparica, Portugal; b Centro de Investigação Interdisciplinar Egas Moniz (CiiEM), Egas Moniz Cooperativa de Ensino Superior, Caparica, Portugal; c Pratica Clínica Privada; d Instituto Universitário Egas Moniz (IUEM), Egas Moniz Cooperativa de Ensino Superior, Caparica, Portugal

## Abstract

**Introduction:**

Temporomandibular disorders have a considerable prevalence, with a significant impact on physical and psychosocial factors [1]. It contributes to high socioeconomic costs, which are generally associated with comorbidities such as depression and other psychological factors [2,3]. The Diagnostic Criteria for Temporomandibular Disorders is based on a bi-mechanical model of pain with two axes: physical signs and symptoms (axis I) and psychological factors (axis II). Psychological factors such as catastrophizing pain, psychic distress, guiding beliefs, beliefs related to painful perception, depressed or anxious mood, and passive coping are all related to an increased pain perception, increased levels of disability, in patients with chronic pain with temporomandibular disorders [3,5]. Psychosocial factors were also identified as predictors of treatment outcome in patients with temporomandibular disorders [6]. We consider that somatic awareness is an important sensory-discriminative factor to be taken into account in this group of patients. The aim of this case study is to analyse if the identification of psychosocial aspects contributes in controlling the temporomandibular symptoms.

**Material and methods:**

A case study of a 25-year-old female patient with generalised headache, generalised myofascial pain (III) in the head and neck region, self-reported awake and sleep bruxism and important psychological factors related to catastrophic pain and anxiety, all described during the initial interview. The patient can positively correlate beginning of the manifestation of the painful symptoms with relevant psychosocial and drastic, in her own words, family changes. Pain intensity and headache are measured with numeric pain rating scale (NPRS) and the patient is submitted to one session of physical therapy per week. The treatment plan consisted of cognitive-behavioral therapy with a first appointment based on education, habits recognition and modification, the patient was medicated with muscle relaxants and off label gabapentine, physiotherapy and psychotherapy. All the assumptions of the Helsinki Declaration have been fulfilled and an informed consent for clinical case of Clinica Dentária Egas Moniz approved by the ethic commission of Instituto Universitário Egas Moniz.

**Results:**

Three months after (12 sessions of physical therapy) the beginning of the treatment plan, the biggest breakthrough was the ability of the patient to identify psychological situations that trigger the exacerbation of pain. The patient referred absence of headache, a significant reduction of myofascial pain (I) located in the masseter and in the temporal of about 70% of the pain scale and changed from myofascial pain to local myalgia.

**Discussion and conclusion:**

Psychosocial factors are frequently present in patients with temporomandibular disorders and their evaluation, grading and consequent intervention become important for the prognosis and resolution of the case.The assessment of psychosocial aspects should be considered in all patients with temporomandibular disorders in order to analyse, case by case, whether they are relevant for controlling their symptoms. It will be important to carry out a experimental study with a larger sample to verify whether the obtained results point in the same direction.
